# Sanitary and Statistical Report of the Surgeon-General of the Navy for the Year 1881

**Published:** 1884-02

**Authors:** 


**Sanitary and Statistical Report of the Surgeon-General of the Navy for the Year 1881.** Washington: Government Printing House. 1883.

The present volume is the usual report issued by the medical department of the navy. Its contents are valuable to the profession. We have a criticism to make in regard to the binding: Such a work should be presented in better dress. Our government can well afford to send forth the reports of investigations of disease, of the able body which comprises its medical and surgical staff, in a style of binding and paper which would raise them above the form in which the political speeches of members of Congress are usually presented.

